# Supplementary material for: Coral reefs in the Gilbert Islands of Kiribati: Resistance, resilience, and recovery after more than a decade of multiple stressors
Source: PLoS One. 2021 Aug 11;16(8):e0255304. doi: 10.1371/journal.pone.0255304 (PMC8357116; doi:10.1371/journal.pone.0255304)
Supplement: S1 Table — (DOCX) [file pone.0255304.s001.docx]

**S1 Table. Environmental variable, coordinates, and years we collected data for 19 study sites visited between 2012 and 2018.**

| **Atoll** | **Site** | **Years Visited**  **(20__)** | | | | **Lat** | **Long** | **Exposure Metric^1^** | **Mean NDV^I2^** | **Population Metric^3^** | **CV_SST_^4^** | **Mean Chl-a (**mg m^3^) | **# of CoTs scars (2014)** |
| --- | --- | --- | --- | --- | --- | --- | --- | --- | --- | --- | --- | --- | --- |
|  |  | **12** | **14** | **16** | **18** |  |  |  |  |  |  |  |  |
| Abaiang | ABG001 |  |  |  |  | 1.8577 | 172.8796 | 0.48 | 0.33 | 4.07 | 3.24 | 0.57 | 18 |
|  | ABG002 |  |  |  |  | 1.8820 | 172.8180 | 0.60 | 0.41 | 5.95 | 3.23 | 0.19 | 0 |
|  | ABG003 |  |  |  |  | 1.7143 | 172.9865 | 0.46 | 0.38 | 5.29 | 3.20 | 0.57 | 85 |
|  | ABG004 |  |  |  |  | 1.8626 | 172.8802 | 0.17 | 0.35 | 4.09 | 3.33 | 0.19 | 81 |
|  | ABG005 |  |  |  |  | 1.9209 | 172.8039 | 0.23 | 0.38 | 4.89 | 3.16 | 0.17 | -- |
|  | ABG006 |  |  |  |  | 1.8972 | 172.7772 | 0.15 | 0.35 | 4.98 | 3.20 | 0.17 | -- |
|  | ABG010 |  |  |  |  | 1.8784 | 172.8463 | 0.50 | 0.45 | 4.74 | 3.17 | 1.03 | 8 |
|  | ABG011 |  |  |  |  | 1.8033 | 172.9088 | 0.26 | 0.36 | 3.37 | 3.24 | 0.29 | 1 |
|  | TRW005 |  |  |  |  | 1.6325 | 172.9673 | 0.74 | 0.21 | 5.55 | 3.31 | 0.17 | 29 |
|  | TRW007 |  |  |  |  | 1.6178 | 172.9336 | 0.63 | 0.27 | 5.67 | 3.18 | 0.15 | 0 |
| Tarawa | TRW002 |  |  |  |  | 1.3332 | 173.0217 | 0.13 | 0.30 | 7.42 | 3.27 | 0.17 | 0 |
|  | TRW008 |  |  |  |  | 1.3581 | 173.1446 | 0.59 | 0.26 | 7.60 | 3.28 | 0.27 | -- |
|  | TRW010 |  |  |  |  | 1.3302 | 172.9634 | 0.48 | 0.11 | 7.56 | 3.30 | 0.17 | 6 |
|  | TRW011 |  |  |  |  | 1.3570 | 173.0790 | 0.70 | 0.26 | 8.73 | 3.31 | 0.18 | -- |
|  | TRW012 |  |  |  |  | 1.3246 | 172.9951 | 0.66 | 0.19 | 7.42 | 3.29 | 0.17 | -- |
|  | TRW013 |  |  |  |  | 1.3463 | 172.9241 | 0.64 | 0.26 | 9.47 | 3.31 | 0.23 | 0 |
|  | TRW014 |  |  |  |  | 1.4209 | 172.9146 | 0.26 | 0.45 | 7.81 | 3.23 | 0.39 | 0 |
|  | TRW015 |  |  |  |  | 1.3506 | 173.0466 | 0.73 | 0.26 | 8.56 | 3.34 | 0.18 | -- |
|  | TRW016 |  |  |  |  | 1.3911 | 173.1507 | 0.54 | 0.31 | 7.40 | 3.32 | 0.16 | -- |

^1^Metric calculated using the prevailing wind direction and a compass heading perpendicular to the reef crest (see Wind and Wave Exposure). Lower values are more sheltered, while higher values are more exposed.

^2^A human disturbance metric using the National Difference Vegetation Index (see Human disturbance). These values range from -1.0 to 1.0, with higher values equal to lower human disturbance.

^3^A human disturbance metric using the distance of each site to the nearest village divided by the population of that village (see Human disturbance). Higher values equal greater human disturbance.

^4^Coefficient of variation for daily Sea Surface Temperature (from 1985 – 2018; see Oceanographic Data).
