# Supplementary material for: Coral reefs in the Gilbert Islands of Kiribati: Resistance, resilience, and recovery after more than a decade of multiple stressors
Source: PLoS One. 2021 Aug 11;16(8):e0255304. doi: 10.1371/journal.pone.0255304 (PMC8357116; doi:10.1371/journal.pone.0255304)
Supplement: S2 Table — (DOCX) [file pone.0255304.s002.docx]

**S2 Table. Coral order, family, and species observed in Tarawa and Abaiang Atolls (as reported by Lovell et al, 2000).**

| **Class & Order** | **Family** | **Species** |
| --- | --- | --- |
| Class ANTHOZOA, subclass ZOOANTHERIA, order SCLERACTINIA |  |  |
|  | THAMNASTERIIDAE |  |
|  |  | *Psammocora (P.) haimeana* (Edwards and Haime, 1851) |
|  |  | *Psammocora profundacella* (Gardiner, 1898) |
|  | POCILLOPORIDAE |  |
|  |  | *Pocillopora damicornis* (Linnaeus, 1758) |
|  |  | *Pocillopora eydouxi* (Edwards & Haime, 1860) |
|  |  | *Seriatopora hystrix* (Dana, 1846) |
|  |  | *Stylophora pistallata* (Esper, 1797) |
|  | ACROPORIDAE |  |
|  |  | *Acropora (A.) abrotanoides* (Lamark, 1816) |
|  |  | *Acropora (A.) anthrocercis* (Brook, 1891) |
|  |  | *Acropora (A.) cerealis* (Dana, 1846) |
|  |  | *Acropora (A.) digitifera* (Dana, 1846) |
|  |  | *Acropora (A.) divaricate* (Dana, 1846) |
|  |  | *Acropora (A.) echinate* (Dana, 1846) |
|  |  | *Acropora (A.) gemmifera* (Brook, 1892) |
|  |  | *Acropora (A.) grandis* (Brook, 1892*)* |
|  |  | *Acropora (A.) humilis* (Dana, 1846) |
|  |  | *Acropora (A.) hyacinthus* (Dana, 1846) |
|  |  | *Acropora (A.) intermedia* (Dana, 1846) |
|  |  | *Acropora (A.) latistella* (Brook, 1892) |
|  |  | *Acropora (A.) lovelli* (Veron and Wallace, 1984) |
|  |  | *Acropora (A.) microphthalma* (Verrill, 1869) |
|  |  | *Acropora (A.) muricata* (Dana, 1846) |
|  |  | *Acropora (A.) nana* (Studer, 1878) |
|  |  | *Acropora (A.) nasuta* (Dana, 1846) |
|  |  | *Acropora (A.) paniculata* (Verrill, 1902) |
|  |  | *Acropora (A.) robusta* (Dana, 1846) |
|  |  | *Acropora (A.) secale* (Studer, 1878) |
|  |  | *Acropora (A.) selago* (Studer, 1878) |
|  |  | *Acropora (A.) spicifera* (Dana, 1846) |
|  |  | *Acropora (A.) tenuis* (Dana, 1846) |
|  |  | *Acropora (A.) tortuosa* (Dana, 1846) |
|  |  | *Acropora (A.) valida* (Dana, 1846) |
|  |  | *Acropora (A.) verweyi* (Veron and Wallace, 1984) |
|  |  | *Acropora (A.) sp.* |
|  |  | *Astreopora listeri* (Bernard, 1896) |
|  |  | *Astreopora myriopthalma* (Lamark 1816) |
|  |  | *Montipora efflorescens* (Bernard, 1897) |
|  |  | *Montipora foveolate* (Dana, 1846) |
|  |  | *Montipora grisea* (Bernard, 1897) |
|  |  | *Montipora hispida* (Dana, 1846)6) |
|  |  | *Montipora hoffmeisteri* (Wells, 1954) |
|  |  | *Montipora informis* (Bernard, 1897) |
|  |  | *Montipora peltiformis* (Bernard, 1897) |
|  |  | *Montipora terbuculosa* (Lamarck, 1816) |
|  |  | *Montipora venosa* (Ehrenberg, 1834) |
|  |  | *Montipora verrucose* (Lamarck, 1816) |
|  | AGARICIIDAE |  |
|  |  | *Gardineroseris planulata* (Dana, 1846) |
|  |  | *Leptoseris mycetoseroides* (Wells, 1954) |
|  |  | *Pachyseris speciosa* (Dana, 1846) |
|  |  | *Pavona cactus* (Forskal, 1775) |
|  |  | *Pavona clavus* (Dana, 1846) |
|  |  | *Pavona explanulata* (Lamarck, 1816) |
|  |  | *Pavona maldivensis* (Gardiner, 1905) |
|  |  | *Pavona minuta* (Wells, 1954) |
|  |  | *Pavona varians* (Verrill, 1864) |
|  | SIDERASTREIDAE |  |
|  |  | *Coscinaraea columna* (Dana, 1846) |
|  | FUNGIIDAE |  |
|  |  | *Cycloseris costulata* |
|  |  | *Fungia (D.) horrida* (Dana, 1846) |
|  |  | *Fungia (D.) valida* (Verrill, 1864) |
|  |  | *Fungia (F.) fungites* (Linnaeus, 1758) |
|  |  | *Fungia (P.) scutaria* (Lamarck, 1801) |
|  |  | *Fungia (V.) concinna* (Verrill, 1864) |
|  |  | *Fungia (V.) granulosa* (Klunzinger, 1879) |
|  |  | *Fungia (V.) repanda* (Dana, 1846) |
|  |  | *Halomitra pileus* (Linnaeus, 1758) |
|  |  | *Herpolitha limax* (Houttuyn, 1772) |
|  |  | *Podobacia crustacea* (Edwards and Haime, 1849) |
|  |  | *Sandalolitha robusta* (Quelch, 1886) |
|  | PORITIDAE |  |
|  |  | *Goniopora stutchburyi* (Wells, 1955) |
|  |  | *Gonipora sp.* |
|  |  | *Porites (P.) cylindrica* (Dana, 1846) |
|  |  | *Porites (P.) lichen* (Dana, 1846) |
|  |  | *Porites (P.) lobata* (Dana, 1846) |
|  |  | *Porites (P.) lutea* (Edward & Haime, 1860) |
|  |  | *Porites rus* (Forskal, 1775) |
|  |  | *Porites (S.) rus* (Forskal, 1775) |
|  |  | *Porites sp.* |
|  | FAVIIDAE |  |
|  |  | *Cyphastrea microphthalma* (Lamarck, 1816) |
|  |  | *Cyphastrea serailia* (Forskal, 1775) |
|  |  | *Echinopora horrida* (Dana, 1846) |
|  |  | *Echinopora lamellosa* (Esper, 1795) |
|  |  | *Favia favus* (Forskal, 1775) |
|  |  | *Favia matthaii* (Vaughan, 1918) |
|  |  | *Favia pallida* (Dana, 1846) |
|  |  | *Favia rotumana* (Gardiner, 1899) |
|  |  | *Favia stelligera* (Dana, 1846) |
|  |  | *Favites chinensis* (Verrill, 1866) |
|  |  | *Favites flexuosa* (Dana, 1846) |
|  |  | *Favites pentagona* (Esper, 1794) |
|  |  | *Favites russelli* (Wells, 1954) |
|  |  | *Goniastrea aspera* |
|  |  | *Goniastrea edwardsi* (Chevalier, 1971) |
|  |  | *Goniastrea favulus* (Dana, 1846) |
|  |  | *Goniastrea pectinate* (Ehrenberg, 1834) |
|  |  | *Leptastrea bewickensis* (Veron, Pinchon, and Wijsman-best, 1977) |
|  |  | *Leptastrea pruinosa* (Crossland, 1952) |
|  |  | *Leptastrea purpurea* (Dana, 1846) |
|  |  | *Leptastrea sp.* |
|  |  | *Leptoria phrygia* (Ellis & Solander, 1786) |
|  |  | *Montastrea curta* (Dana, 1846) |
|  |  | *Montastrea magnistellata* (Chevalier, 1971) |
|  |  | *Oulophyllia crispa* (Lamarck, 1816) |
|  |  | *Platygyra daedalea* (Ellis & Solander, 1786) |
|  |  | *Platygyra sinensis* (Edward & Haime, 1849) |
|  | MERULINIDAE |  |
|  |  | *Hydnophora exesa* (Pallas, 1766) |
|  |  | *Hydnophora microconos* (Lamarck, 1816) |
|  |  | *Hydnophora rigida* (Dana, 1846) |
|  |  | *Merulina ampliata* (Ellis & Solander, 1786) |
|  | MUSSIDAE |  |
|  |  | *Acanthastrea echinate* (Dana, 1846) |
|  |  | *Lobophyllia corymbose* (Forskal, 1775) |
|  |  | *Lobophyllia hemprichii* (Ehrenberg, 1834) |
|  |  | *Symphillia radians* (Edwards & Haime, 1849) |
|  | PECTINIIDAE |  |
|  |  | *Echinophyllia echinata* (Saville-Kent, 1871) |
|  |  | *Echinophyllia sp.* |
|  |  | *Mycedium elephantotos* (Pallas, 1766) |
|  |  | *Oxypora lacera* (Verrill, 1864) |
|  | CARYPHYLLIDAE |  |
|  |  | *Plerogyra simplex* (Rehberg, 1892) |
|  | DENDROPHYLLIIDAE |  |
|  |  | *Tubastrea micrantha* (Ehrenberg, 1834) |
|  |  | *Turbinaria frondens* (Dana, 1846) |
|  |  | *Turbinaria mesenterina* |
|  |  | *Turbinaria reinformis* (Bernard, 1896) |
|  |  | *Turbinaria sp.* |
| Class HYDROZOA, order COENOTHECALIA |  |  |
|  | HELIOPORIDAE |  |
|  |  | *Heliopora coerulea* (Pallas, 1766) |
| Order MILLEPORINA |  |  |
|  | MILLEPORIDAE |  |
|  |  | *Millepora platyphylla* (Hemprich & Ehrenberg, 1834) |
|  |  | *Millepora sp.* |
|  | STYLASTERIDAE |  |
|  |  | *Distochopora violacea* (Pallas, 1776) |
