# Supplementary material for: Coral reefs in the Gilbert Islands of Kiribati: Resistance, resilience, and recovery after more than a decade of multiple stressors
Source: PLoS One. 2021 Aug 11;16(8):e0255304. doi: 10.1371/journal.pone.0255304 (PMC8357116; doi:10.1371/journal.pone.0255304)
Supplement: S3 Table — Statistically significant results at α = 0.05 are in bold, while those that are significant at α = 0.10 are underlined. (DOCX) [file pone.0255304.s003.docx]

**S3 Table. Results of linear mixed effects models for each key benthic category, including additional LMM for subsets of the data.** Statistically significant results at α = 0.05 are in bold, while those that are significant at α = 0.10 are underlined.

|  | ***All Sites*** | | | | | ***Sites affected by CoTs*** | | | | | ***Sites Visited Every Year*** | | | |
| --- | --- | --- | --- | --- | --- | --- | --- | --- | --- | --- | --- | --- | --- | --- |
| **Categories** | **χ^2^** | **p** | **Marg  R^2^** | **Cond R^2^** | **χ^2^** | | **p** | **Marg R^2^** | **Cond R^2^** | **χ^2^** | | **p** | **Marg R^2^** | **Cond R^2^** |
| ***Hard Coral Taxa*** | | | | | | | | | | | | | | |
| All Live Coral | **8.36** | **0.04** | **0.03** | **0.89** | 3.29 | | 0.35 | 0.04 | 0.77 | 6.76 | | 0.08 | 0.08 | 0.82 |
| *Acropora* | 7.74 | 0.05 | 0.18 | 0.19 | 4.04 | | 0.26 | 0.19 | 0.19 | 6.94 | | 0.07 | 0.26 | 0.26 |
| Favids | **15.00** | **<0.01** | **0.32** | **0.49** | **5.74** | | **0.12** | **0.07** | **0.07** | **10.44** | | **0.02** | **0.37** | **0.53** |
| *Heliopora* | **8.59** | **0.04** | **0.05** | **0.79** | **8.01** | | **0.05** | **0.11** | **0.83** | **11.07** | | **0.01** | **0.13** | **0.82** |
| *Montipora* | **16.41** | **<0.01** | **0.35** | **0.45** | **11.61** | | **0.01** | **0.44** | **0.56** | **6**.**73** | | **0.08** | **0.25** | **0.29** |
| *Pocillopora* | **14.77** | **<0.01** | **0.14** | **0.68** | 6.16 | | 0.10 | 0.22 | 0.50 | 6.88 | | 0.08 | 0.19 | 0.48 |
| *Porites* (Massive) | **9.62** | **0.02** | **0.07** | **0.79** | 6.53 | | 0.09 | 0.07 | 0.85 | 7.31 | | 0.06 | 0.05 | 0.86 |
| *P. rus* | 6.75 | 0.08 | 0.02 | 0.91 | 3.15 | | 0.37 | 0.05 | 0.73 | 3.21 | | 0.36 | 0.03 | 0.81 |
| ***Macroalgae Taxa*** | | | | | | | | | | | | | | |
| All Macroalgae | **19.68** | **<0.01** | **0.29** | **0.53** | **9.73** | | **0.02** | **0.44** | **0.55** | **13.00** | | **<0.01** | **0.40** | **0.63** |
| *Halimeda* | **14.27** | **<0.01** | **0.26** | **0.55** | **10.02** | | **0.02** | **0.36** | **0.52** | **12.77** | | **0.01** | **0.32** | **0.62** |
| *Lobophora* | 5.15 | 0.16 | 0.09 | 0.33 | 5.28 | | 0.15 | 0.19 | 0.47 | 1.98 | | 0.58 | 0.07 | 0.24 |
| ***Other Benthic Categories*** | | | | | | | | | | | | | | |
| CCA* | **7.95** | **0.05** | **0.14** | **0.38** | 5.69 | | 0.13 | 0.12 | 0.68 | 4.35 | | 0.23 | 0.16 | 0.26 |
| Corallimorphs | 4.06 | 0.26 | 0.12 | 0.51 | 3.86 | | 0.28 | 0.17 | 0.52 | 5.52 | | 0.14 | 0.15 | 0.50 |
| Cyanobacteria | 5.21 | 0.16 | 0.08 | 0.41 | 4.35 | | 0.23 | 0.20 | 0.23 | 2.41 | | 0.49 | 0.10 | 0.26 |
| Rubble | **9.81** | **0.02** | **0.14** | **0.53** | 5.87 | | 0.12 | 0.26 | 0.26 | 6.21 | | 0.10 | 0.20 | 0.50 |
| Sand | 6.33 | 0.10 | 0.06 | 0.71 | 2.74 | | 0.43 | 0.08 | 0.57 | 2.20 | | 0.53 | 0.05 | 0.59 |
| Soft Coral | 1.48 | 0.69 | 0.04 | 0.40 | 5.16 | | 0.16 | 0.17 | 0.57 | 1.40 | | 0.70 | 0.04 | 0.63 |
| Sponges | **14.09** | **<0.01** | **0.16** | **0.64** | **18.22** | | **<0.01** | **0.63** | **0.63** | **11.51** | | **0.01** | **0.34** | **0.63** |
| Turf algae | **14.34** | **<0.01** | **0.17** | **0.66** | **14.92** | | **<0.01** | **0.28** | **0.82** | **11.64** | | **0.01** | **0.28** | **0.70** |
