# Supplementary material for: Coral reefs in the Gilbert Islands of Kiribati: Resistance, resilience, and recovery after more than a decade of multiple stressors
Source: PLoS One. 2021 Aug 11;16(8):e0255304. doi: 10.1371/journal.pone.0255304 (PMC8357116; doi:10.1371/journal.pone.0255304)
Supplement: S4 Table — All p-values have been adjusted for multiple comparisons. Results significant at sigma = 0.05 are in bold; those significant at sigma = 0.10 are underlined. (DOCX) [file pone.0255304.s004.docx]

**S4 Table. Turkey results for Linear Mixed Effects Models (Percent ~ Year + (1|Site).**All p-values have been adjusted for multiple comparisons. Results significant at sigma = 0.05 are in bold; those significant at sigma = 0.10 are underlined.

***Model results for dataset including all sites.***

**Hard coral taxa**

All live hard coral

| **Years** | **Estimate** | **St. Error** | **z-value** | **p-value** |
| --- | --- | --- | --- | --- |
| **2012 – 2014** | **-7.589** | **2.768** | **-2.741** | **0.031** |
| 2012 – 2016 | -6.497 | 2.644 | -2.446 | 0.068 |
| 2012 – 2018 | -5.382 | 2.535 | -2.123 | 0.144 |
| 2014 – 2016 | 1.111 | 2.385 | 0.466 | 0.966 |
| 2014 – 2018 | 2.196 | 2.341 | 0.938 | 0.783 |
| 2016 – 2018 | 1.085 | 2.178 | 0.498 | 0.959 |

*Acropora*

| **Years** | **Estimate** | **St. Error** | **z-value** | **p-value** |
| --- | --- | --- | --- | --- |
| 2012 – 2014 | 0.464 | 0.268 | 1.731 | 0.307 |
| 2012 – 2016 | -0.221 | 0.277 | -0.798 | 0.855 |
| 2012 – 2018 | -0.119 | 0.245 | -0.485 | 0.962 |
| 2014 – 2016 | -0.685 | 0.277 | -2.470 | 0.065 |
| 2014 – 2018 | -0.583 | 0.244 | -2.382 | 0.080 |
| 2016 – 2018 | 0.103 | 0.255 | 0.402 | 0.978 |

Favids

| **Years** | **Estimate** | **St. Error** | **z-value** | **p-value** |
| --- | --- | --- | --- | --- |
| 2012 – 2014 | -1.125 | 0.479 | -2.349 | 0.086 |
| 2012 – 2016 | -1.132 | 0.478 | -2.366 | 0.083 |
| 2012 – 2018 | 0.270 | 0.442 | 0.611 | 0.928 |
| 2014 – 2016 | -0.007 | 0.457 | -0.014 | 1.000 |
| **2014 – 2018** | **1.395** | **0.420** | **-3.322** | **0.005** |
| **2016 – 2018** | **1.402** | **0.415** | **3.375** | **0.004** |

*Heliopora*

| **Years** | **Estimate** | **St. Error** | **z-value** | **p-value** |
| --- | --- | --- | --- | --- |
| 2012 – 2014 | -0.089 | 0.969 | -0.092 | 1.000 |
| 2012 – 2016 | -1.893 | 0.924 | -2.050 | 0.169 |
| 2012 – 2018 | -1.895 | 0.912 | -2.077 | 0.160 |
| 2014 – 2016 | -1.804 | 0.833 | -2.165 | 0.132 |
| 2014 – 2018 | -1.806 | 0.819 | -2.204 | 0.121 |
| 2016 – 2018 | -0.002 | 0.780 | -0.002 | 1.000 |

*Montipora*

| **Years** | **Estimate** | **St. Error** | **z-value** | **p-value** |
| --- | --- | --- | --- | --- |
| 2012 – 2014 | -0.089 | 0.126 | -0.711 | 0.892 |
| 2012 – 2016 | -0.205 | 0.116 | -1.764 | 0.289 |
| 2012 – 2018 | 0.212 | 0.113 | 1.885 | 0.233 |
| 2014 – 2016 | -0.116 | 0.116 | -0.996 | 0.750 |
| **2014 – 2018** | **0.301** | **0.112** | **2.704** | **0.035** |
| **2016 – 2018** | **0.417** | **0.100** | **4.182** | **<0.001** |

*Pocillopora*

| **Years** | **Estimate** | **St. Error** | **z-value** | **p-value** |
| --- | --- | --- | --- | --- |
| 2012 – 2014 | -0.219 | 0.391 | -0.560 | 0.943 |
| 2012 – 2016 | -0.213 | 0.376 | -0.565 | 0.942 |
| 2012 – 2018 | 0.840 | 0.356 | 2.344 | 0.087 |
| 2014 – 2016 | 0.007 | 0.338 | 0.020 | 1.000 |
| 2014 – 2018 | **1.059** | **0.236** | **3.249** | **0.006** |
| 2016 – 2018 | **1.053** | **0.307** | **3.429** | **0.003** |

*Porites* (Massive)

| **Years** | **Estimate** | **St. Error** | **z-value** | **p-value** |
| --- | --- | --- | --- | --- |
| 2012 – 2014 | -0.893 | 0.650 | -1.373 | 0.515 |
| **2012 – 2016** | **-1.920** | **0.640** | **-3.000** | **0.014** |
| 2012 – 2018 | -1.424 | 0.611 | -2.330 | 0.091 |
| 2014 – 2016 | -1.027 | 0.573 | -1.794 | 0.275 |
| 2014 – 2018 | -0.532 | 0.563 | -0.945 | 0.780 |
| 2016 – 2018 | 0.496 | 0.547 | 0.906 | 0.801 |

*Porites rus*

| **Years** | **Estimate** | **St. Error** | **z-value** | **p-value** |
| --- | --- | --- | --- | --- |
| 2012 – 2014 | -5.582 | 2.835 | -1.969 | 0.199 |
| 2012 – 2016 | 0.111 | 2.681 | 0.041 | 1.000 |
| 2012 – 2018 | -0.047 | 2.569 | -0.018 | 1.000 |
| 2014 – 2016 | 5.693 | 2.530 | 2.250 | 0.110 |
| 2014 – 2018 | 5.536 | 2.492 | 2.221 | 0.117 |
| 2016 – 2018 | -0.158 | 2.287 | -0.069 | 1.000 |

**Macroalgae taxa**

All macroalgae genera

| **Years** | **Estimate** | **St. Error** | **z-value** | **p-value** |
| --- | --- | --- | --- | --- |
| **2012 – 2014** | **-14.735** | **3.528** | **-4.177** | **<0.001** |
| **2012 – 2016** | **-15.528** | **3.436** | **-4.519** | **<0.001** |
| **2012 – 2018** | **-11.729** | **3.334** | **-3.518** | **0.002** |
| 2014 – 2016 | -0.793 | 3.018 | -0.263 | 0.994 |
| 2014 – 2018 | 3.006 | 2.888 | 1.041 | 0.724 |
| 2016 – 2018 | 3.799 | 2.771 | 1.371 | 0.515 |

*Halimeda*

| **Years** | **Estimate** | **St. Error** | **z-value** | **p-value** |
| --- | --- | --- | --- | --- |
| 2012 – 2014 | **-13.990** | **4.470** | **-3.130** | **0.009** |
| 2012 – 2016 | **-19.125** | **4.611** | **-4.147** | **<0.001** |
| 2012 – 2018 | **-12.950** | **4.435** | **-2.920** | **0.018** |
| 2014 – 2016 | -5.134 | 4.024 | -1.276 | 0.577 |
| 2014 – 2018 | 1.040 | 3.805 | 0.273 | 0.993 |
| 2016 – 2018 | 6.175 | 4.053 | 1.523 | 0.422 |

*Lobophora*

| **Years** | **Estimate** | **St. Error** | **z-value** | **p-value** |
| --- | --- | --- | --- | --- |
| 2012 – 2014 | -3.506 | 1.995 | -1.757 | 0.292 |
| 2012 – 2016 | -0.132 | 1.884 | -0.070 | 1.000 |
| 2012 – 2018 | -0.319 | 1.837 | -0.168 | 0.998 |
| 2014 – 2016 | 3.374 | 1.749 | 1.929 | 0.214 |
| 2014 – 2018 | 3.197 | 1.710 | 1.869 | 0.240 |
| 2016 – 2018 | -0.177 | 1.547 | -0.114 | 0.999 |

**Other benthic taxa**

*Crustose-coralline algae*

| **Years** | **Estimate** | **St. Error** | **z-value** | **p-value** |
| --- | --- | --- | --- | --- |
| 2012 – 2014 | 3.678 | 1.698 | 2.166 | 0.131 |
| **2012 – 2016** | **4.529** | **1.651** | **2.743** | **0.031** |
| 2012 – 2018 | 3.270 | 1.558 | 2.099 | 0.152 |
| 2014 – 2016 | 0.850 | 1.482 | 0.573 | 0.940 |
| 2014 – 2018 | -0.409 | 1.393 | -0.293 | 0.991 |
| 2016 – 2018 | -1.259 | 1.335 | -0.943 | 0.780 |

*Cyanobacteria*

| **Years** | **Estimate** | **St. Error** | **z-value** | **p-value** |
| --- | --- | --- | --- | --- |
| 2012 – 2014 | 0.186 | 1.778 | 0.104 | 1.000 |
| 2012 – 2016 | -0.346 | 1.724 | -0.200 | 0.997 |
| 2012 – 2018 | -2.584 | 1.630 | -1.585 | 0.385 |
| 2014 – 2016 | -0.531 | 1.547 | -0.343 | 0.986 |
| 2014 – 2018 | -2.770 | 1.463 | -1.893 | 0.229 |
| 2016 – 2018 | -2.239 | 1.396 | -1.604 | 0.374 |

*Rubble*

| **Years** | **Estimate** | **St. Error** | **z-value** | **p-value** |
| --- | --- | --- | --- | --- |
| 2012 – 2014 | **3.919** | **1.406** | **2.787** | **0.027** |
| 2012 – 2016 | 2.249 | 1.360 | 1.654 | 0.346 |
| 2012 – 2018 | 0.899 | 1.289 | 0.698 | 0.897 |
| 2014 – 2016 | -1.167 | 1.220 | -1.369 | 0.517 |
| 2014 – 2018 | **-1.302** | **1.161** | **-2.600** | **0.045** |
| 2016 – 2018 | -1.350 | 1.103 | -1.224 | 0.610 |

*Sand*

| **Years** | **Estimate** | **St. Error** | **z-value** | **p-value** |
| --- | --- | --- | --- | --- |
| 2012 – 2014 | 4.490 | 2.919 | 1.538 | 0.411 |
| 2012 – 2016 | -0.949 | 2.904 | -0.327 | 0.988 |
| 2012 – 2018 | -0.004 | 2.854 | -0.001 | 1.000 |
| 2014 – 2016 | -5.439 | 2.368 | -2.297 | 0.097 |
| 2014 – 2018 | -4.494 | 2.292 | -1.961 | 0.200 |
| 2016 – 2018 | 0.945 | 2.231 | 0.424 | 0.974 |

*Soft Coral*

| **Years** | **Estimate** | **St. Error** | **z-value** | **p-value** |
| --- | --- | --- | --- | --- |
| 2012 – 2014 | 0.097 | 0.101 | 0.956 | 0.774 |
| 2012 – 2016 | 0.101 | 0.101 | 0.998 | 0.750 |
| 2012 – 2018 | 0.062 | 0.094 | 0.658 | 0.912 |
| 2014 – 2016 | 0.004 | 0.101 | 0.042 | 1.000 |
| 2014 – 2018 | -0.035 | 0.094 | -0.373 | 0.982 |
| 2016 – 2018 | -0.039 | 0.904 | -0.419 | 0.975 |

*Sponges*

| **Years** | **Estimate** | **St. Error** | **z-value** | **p-value** |
| --- | --- | --- | --- | --- |
| **2012 – 2014** | **-2.409** | **0.708** | **-3.404** | **0.004** |
| 2012 – 2016 | -0.453 | 0.682 | -0.664 | 0.910 |
| 2012 – 2018 | -0.710 | 0.648 | -1.096 | 0.690 |
| **2014 – 2016** | **1.956** | **0.612** | **3.193** | **0.007** |
| **2014 – 2018** | **1.698** | **0.588** | **2.891** | **0.020** |
| 2016 – 2018 | -0.257 | 0.555 | -0.464 | 0.967 |

*Turf algae*

| **Years** | **Estimate** | **St. Error** | **z-value** | **p-value** |
| --- | --- | --- | --- | --- |
| 2012 – 2014 | **16.882** | **5.035** | **3.353** | **0.004** |
| 2012 – 2016 | **18.669** | **4.850** | **3.849** | **<0.001** |
| 2012 – 2018 | **13.382** | **4.613** | **2.901** | **0.019** |
| 2014 – 2016 | 1.787 | 4.356 | 0.410 | 0.976 |
| 2014 – 2018 | -3.500 | 4.184 | -0.836 | 0.836 |
| 2016 – 2018 | -5.290 | 3.950 | -1.339 | 0.536 |

***Model results for dataset containing only the sites that were affected by COTs (ABG001, ABG003, ABG004, ABG010, ABG011, TRW005, TRW010).***

**Hard coral taxa**

*All live hard coral*

| **Years** | **Estimate** | **St. Error** | **z-value** | **p-value** |
| --- | --- | --- | --- | --- |
| 2012 – 2014 | -5.371 | 2.751 | -1.953 | 0.206 |
| 2012 – 2016 | -6.719 | 2.847 | -2.360 | 0.085 |
| 2012 – 2018 | -3.127 | 2.751 | -1.137 | 0.666 |
| 2014 – 2016 | -1.348 | 2.751 | -0.490 | 0.961 |
| 2014 – 2018 | 2.244 | 2.503 | 0.896 | 0.806 |
| 2016 – 2018 | 3.593 | 2.751 | 1.306 | 0.558 |

*Acropora*

| **Years** | **Estimate** | **St. Error** | **z-value** | **p-value** |
| --- | --- | --- | --- | --- |
| 2012 – 2014 | 0.551 | 0.417 | 1.321 | 0.549 |
| 2012 – 2016 | -0.111 | 0.457 | -0.242 | 0.995 |
| 2012 – 2018 | 0.028 | 0.417 | 0.067 | 1.000 |
| 2014 – 2016 | -0.662 | 0.417 | -1.586 | 0.385 |
| 2014 – 2018 | -0.523 | 0.373 | -1.402 | 0.497 |
| 2016 – 2018 | 0.139 | 0.417 | 0.332 | 0.987 |

Favids

| **Years** | **Estimate** | **St. Error** | **z-value** | **p-value** |
| --- | --- | --- | --- | --- |
| 2012 – 2014 | **-1.701** | **0.595** | **-2.859** | **0.022** |
| 2012 – 2016 | -1.579 | 0.620 | -2.546 | 0.053 |
| 2012 – 2018 | 0.080 | 0.579 | 0.138 | 0.999 |
| 2014 – 2016 | 0.122 | 0.595 | 0.205 | 0.997 |
| **2014 – 2018** | **1.781** | **0.537** | **3.315** | **0.005** |
| **2016 – 2018** | **1.658** | **0.579** | **2.866** | **0.022** |

*Heliopora*

| **Years** | **Estimate** | **St. Error** | **z-value** | **p-value** |
| --- | --- | --- | --- | --- |
| 2012 – 2014 | 1.348 | 1.178 | 1.144 | 0.662 |
| 2012 – 2016 | -2.312 | 1.218 | -1.898 | 0.228 |
| 2012 – 2018 | -1.396 | 1.178 | -1.185 | 0.636 |
| 2014 – 2016 | **-3.660** | **1.178** | **-3.106** | **0.010** |
| 2014 – 2018 | -2.744 | 1.074 | -2.556 | 0.052 |
| 2016 – 2018 | 0.916 | 1.178 | 0.777 | 0.865 |

*Montipora*

| **Years** | **Estimate** | **St. Error** | **z-value** | **p-value** |
| --- | --- | --- | --- | --- |
| 2012 – 2014 | -0.162 | 0.115 | -1.409 | 0.493 |
| 2012 – 2016 | -0.245 | 0.120 | -2.046 | 0.171 |
| 2012 – 2018 | 0.176 | 0.111 | 1.578 | 0.391 |
| 2014 – 2016 | -0.084 | 0.115 | -0.728 | 0.886 |
| **2014 – 2018** | **0.338** | **0.104** | **3.258** | **0.006** |
| **2016 – 2018** | **0.421** | **0.111** | **3.778** | **<0.001** |

*Pocillopora*

| **Years** | **Estimate** | **St. Error** | **z-value** | **p-value** |
| --- | --- | --- | --- | --- |
| 2012 – 2014 | -0.137 | 0.494 | -0.277 | 0.993 |
| 2012 – 2016 | -0.493 | 0.525 | -0.940 | 0.783 |
| 2012 – 2018 | 1.082 | 0.494 | 2.190 | 0.126 |
| 2014 – 2016 | -0.357 | 0.494 | -0.722 | 0.888 |
| **2014 – 2018** | **1.219** | **0.443** | **2.753** | **0.030** |
| **2016 – 2018** | **1.575** | **0.494** | **3.189** | **0.008** |

*Porites* (massive)

| **Years** | **Estimate** | **St. Error** | **z-value** | **p-value** |
| --- | --- | --- | --- | --- |
| 2012 – 2014 | -1.095 | 0.959 | -1.142 | 0.663 |
| **2012 – 2016** | **-2.556** | **0.987** | **-2.591** | **0.050** |
| 2012 – 2018 | -2.148 | 0.952 | -2.255 | 0.108 |
| 2014 – 2016 | -1.462 | 0.959 | -1.525 | 0.422 |
| 2014 – 2018 | -1.054 | 0.875 | -1.204 | 0.624 |
| 2016 – 2018 | 0.408 | 0.952 | 0.428 | 0.974 |

*Porites rus*

| **Years** | **Estimate** | **St. Error** | **z-value** | **p-value** |
| --- | --- | --- | --- | --- |
| 2012 – 2014 | -3.980 | 4.015 | -0.991 | 0.754 |
| 2012 – 2016 | 1.444 | 4.174 | 0.343 | 0.986 |
| 2012 – 2018 | 1.362 | 4.015 | 0.339 | 0.987 |
| 2014 – 2016 | 5.414 | 4.015 | 1.348 | 0.531 |
| 2014 – 2018 | 5.343 | 3.641 | 1.467 | 0.457 |
| 2016 – 2018 | -0.071 | 4.015 | -0.018 | 1.000 |

**Macroalgae Taxa**

*All Macroalgae*

| **Years** | **Estimate** | **St. Error** | **z-value** | **p-value** |
| --- | --- | --- | --- | --- |
| **2012 – 2014** | **-18.866** | **5.683** | **-3.320** | **0.005** |
| **2012 – 2016** | **-22.636** | **6.053** | **-3.739** | **0.001** |
| **2012 – 2018** | **-18.044** | **5.683** | **-3.175** | **0.008** |
| 2014 – 2016 | -3.770 | 5.683 | -0.663 | 0.911 |
| 2014 – 2018 | 0.822 | 5.089 | 0.162 | 0.998 |
| 2016 – 2018 | 0.459 | 5.683 | 0.808 | 0.850 |

*Halimeda*

| **Years** | **Estimate** | **St. Error** | **z-value** | **p-value** |
| --- | --- | --- | --- | --- |
| **2012 – 2014** | **-18.465** | **5.820** | **3.172** | **0.008** |
| **2012 – 2016** | **-26.728** | **6.204** | **-4.308** | **<0.001** |
| **2012 – 2018** | **-18.595** | **5.820** | **-3.195** | **0.008** |
| 2014 – 2016 | -8.264 | 5.820 | -1.420 | 0.486 |
| 2014 – 2018 | -0.130 | 5.211 | -0.025 | 0.999 |
| 2016 – 2018 | 0.814 | 5.820 | 1.397 | 0.500 |

*Lobophora*

| **Years** | **Estimate** | **St. Error** | **z-value** | **p-value** |
| --- | --- | --- | --- | --- |
| 2012 – 2014 | -0.438 | 1.836 | -0.238 | 0.995 |
| 2012 – 2016 | 3.996 | 1.935 | 2.065 | 0.164 |
| 2012 – 2018 | 0.391 | 1.767 | 0.221 | 0.996 |
| 2014 – 2016 | 4.433 | 1.856 | 2.415 | 0.074 |
| 2014 – 2018 | 0.826 | 1.657 | 0.500 | 0.959 |
| 2016 – 2018 | -3.605 | 1.767 | -2.041 | 0.173 |

**Other Benthic Taxa**

CCA

| **Years** | **Estimate** | **St. Error** | **z-value** | **p-value** |
| --- | --- | --- | --- | --- |
| 2012 – 2014 | 2.879 | 2.057 | 1.399 | 0.499 |
| 2012 – 2016 | 5.522 | 2.161 | 2.556 | 0.052 |
| 2012 – 2018 | 2.689 | 2.057 | 1.307 | 0.558 |
| 2014 – 2016 | 2.643 | 2.057 | 1.285 | 0.572 |
| 2014 – 2018 | -0.190 | 1.853 | -0.103 | 0.999 |
| 2016 – 2018 | -2.833 | 2.057 | -1.377 | 0.513 |

Cyanobacteria

| **Years** | **Estimate** | **St. Error** | **z-value** | **p-value** |
| --- | --- | --- | --- | --- |
| 2012 – 2014 | -1.065 | 1.574 | -0.677 | 0.906 |
| 2012 – 2016 | 0.423 | 1.724 | 0.245 | 0.995 |
| 2012 – 2018 | 0.982 | 1.574 | 0.624 | 0.924 |
| 2014 – 2016 | 1.488 | 1.574 | 0.946 | 0.779 |
| 2014 – 2018 | 2.047 | 1.407 | 1.455 | 0.464 |
| 2016 – 2018 | 0.560 | 1.563 | -0.956 | 0.773 |

Rubble

| **Years** | **Estimate** | **St. Error** | **z-value** | **p-value** |
| --- | --- | --- | --- | --- |
| 2012 – 2014 | 4.049 | 1.821 | 2.223 | 0.117 |
| **2012 – 2016** | **5.361** | **1.929** | **2.779** | **0.028** |
| 2012 – 2018 | 1.521 | 1.821 | 0.835 | 0.837 |
| 2014 – 2016 | 1.312 | 1.821 | 0.721 | 0.889 |
| 2014 – 2018 | -2.528 | 1.634 | -1.547 | 0.408 |
| 2016 – 2018 | -3.841 | 1.821 | -2.109 | 0.150 |

Sand

| **Years** | **Estimate** | **St. Error** | **z-value** | **p-value** |
| --- | --- | --- | --- | --- |
| 2012 – 2014 | 3.469 | 4.029 | 0.861 | 0.825 |
| 2012 – 2016 | 0.295 | 4.248 | 0.069 | 1.000 |
| 2012 – 2018 | -0.369 | 4.029 | -0.092 | 1.000 |
| 2014 – 2016 | -3.174 | 4.029 | -0.788 | 0.860 |
| 2014 – 2018 | -3.837 | 3.622 | -1.059 | 0.714 |
| 2016 – 2018 | -0.664 | 4.029 | -0.165 | 0.998 |

Soft Coral

| **Years** | **Estimate** | **St. Error** | **z-value** | **p-value** |
| --- | --- | --- | --- | --- |
| 2012 – 2014 | 0.186 | 0.110 | 1.697 | 0.325 |
| 2012 – 2016 | 0.191 | 0.110 | 1.745 | 0.300 |
| 2012 – 2018 | 0.077 | 0.107 | 0.721 | 0.889 |
| 2014 – 2016 | 0.005 | 0.110 | 0.048 | 1.000 |
| 2014 – 2018 | -0.109 | 0.107 | -1.018 | 0.739 |
| 2016 – 2018 | -0.114 | 0.107 | -1.067 | 0.709 |

Sponges

| **Years** | **Estimate** | **St. Error** | **z-value** | **p-value** |
| --- | --- | --- | --- | --- |
| 2012 – 2014 | -0.864 | 0.498 | -1.737 | 0.303 |
| 2012 – 2016 | 1.345 | 0.545 | 2.468 | 0.065 |
| 2012 – 2018 | 0.840 | 0.498 | 1.688 | 0.329 |
| **2014 – 2016** | **2.209** | **0.480** | **4.440** | **<0.001** |
| **2014 – 2018** | **1.704** | **0.445** | **3.829** | **<0.001** |
| 2016 – 2018 | -0.505 | 0.498 | -1.015 | 0.740 |

Turf Algae

| **Years** | **Estimate** | **St. Error** | **z-value** | **p-value** |
| --- | --- | --- | --- | --- |
| 2012 – 2014 | 20.539 | 4.232 | 4.853 | <0.001 |
| 2012 – 2016 | 20.476 | 4.392 | 4.662 | <0.001 |
| 2012 – 2018 | 19.157 | 4.232 | 4.527 | <0.001 |
| 2014 – 2016 | -0.063 | 4.232 | -0.015 | 1.000 |
| 2014 – 2018 | -1.382 | 3.843 | -0.360 | 0.984 |
| 2016 – 2018 | -1.319 | 4.232 | -0.312 | 0.989 |

***Model results for dataset containing only the sites that were visited each year (ABG001, ABG002, ABG003, TRW002, and TRW010).***

**Hard coral taxa**

All Live Coral

| **Years** | **Estimate** | **St. Error** | **z-value** | **p-value** |
| --- | --- | --- | --- | --- |
| 2012 – 2014 | -4.812 | 2.989 | -1.500 | 0.438 |
| 2012 – 2016 | -7.642 | 2.989 | -2.557 | 0.052 |
| 2012 – 2018 | -5.220 | 2.989 | -1.747 | 0.300 |
| 2014 – 2016 | -3.160 | 2.989 | -1.057 | 0.716 |
| 2014 – 2018 | -0.739 | 2.989 | -0.247 | 0.995 |
| 2016 – 2018 | 2.422 | 2.989 | 0.810 | 0.850 |

*Acropora*

| **Years** | **Estimate** | **St. Error** | **z-value** | **p-value** |
| --- | --- | --- | --- | --- |
| 2012 – 2014 | 1.145 | 1.679 | 0.682 | 0.904 |
| 2012 – 2016 | -0.131 | 1.860 | -0.071 | 1.000 |
| 2012 – 2018 | 1.164 | 1.754 | 0.663 | 0.911 |
| 2014 – 2016 | -1.277 | 1.790 | -0.713 | 0.892 |
| 2014 – 2018 | 0.018 | 1.679 | 0.011 | 1.000 |
| 2016 – 2018 | 1.295 | 1.860 | 0.696 | 0.898 |

Favids

| **Years** | **Estimate** | **St. Error** | **z-value** | **p-value** |
| --- | --- | --- | --- | --- |
| 2012 – 2014 | -1.322 | 0.526 | -2.514 | 0.058 |
| **2012 – 2016** | **-1.485** | **0.526** | **-2.824** | **0.024** |
| 2012 – 2018 | -0.260 | 0.526 | -0.494 | 0.960 |
| 2014 – 2016 | -0.163 | 0.526 | -0.310 | 0.990 |
| 2014 – 2018 | 1.062 | 0.526 | 2.020 | 0.181 |
| 2016 – 2018 | 1.225 | 0.526 | 2.330 | 0.091 |

*Heliopora*

| **Years** | **Estimate** | **St. Error** | **z-value** | **p-value** |
| --- | --- | --- | --- | --- |
| 2012 – 2014 | -8.171 | 10.197 | -0.801 | 0.853 |
| 2012 – 2016 | -3.580 | 9.038 | -0.396 | 0.979 |
| 2012 – 2018 | 1.067 | 10.197 | 0.105 | 1.000 |
| 2014 – 2016 | 4.591 | 9.038 | 0.508 | 0.957 |
| 2014 – 2018 | 9.238 | 10.197 | 0.906 | 0.801 |
| 2016 – 2018 | 4.647 | 9.038 | 0.514 | 0.955 |

*Montipora*

| **Years** | **Estimate** | **St. Error** | **z-value** | **p-value** |
| --- | --- | --- | --- | --- |
| 2012 – 2014 | -2.064 | 7.489 | -0.276 | 0.993 |
| 2012 – 2016 | -2.779 | 8.204 | -0.339 | 0.987 |
| 2012 – 2018 | 13.862 | 7.783 | 1.781 | 0.282 |
| 2014 – 2016 | -0.714 | 7.489 | -0.095 | 1.000 |
| 2014 – 2018 | 15.926 | 7.025 | 2.267 | 0.105 |
| 2016 – 2018 | 16.640 | 7.783 | 2.138 | 0.141 |

*Pocillopora*

| **Years** | **Estimate** | **St. Error** | **z-value** | **p-value** |
| --- | --- | --- | --- | --- |
| 2012 – 2014 | -3.610 | 7.103 | -0.508 | 0.957 |
| 2012 – 2016 | 2.057 | 5.800 | 0.353 | 0.985 |
| 2012 – 2018 | -4.340 | 6.484 | -0.669 | 0.908 |
| 2014 – 2016 | 5.657 | 7.103 | 0.796 | 0.855 |
| 2014 – 2018 | -0.730 | 7.672 | -0.095 | 1.000 |
| 2016 – 2018 | -6.387 | 6.484 | -0.985 | 0.756 |

*Porites* (massive)

| **Years** | **Estimate** | **St. Error** | **z-value** | **p-value** |
| --- | --- | --- | --- | --- |
| 2012 – 2014 | 11.827 | 8.634 | 1.370 | 0.518 |
| 2012 – 2016 | 11.654 | 8.634 | 1.350 | 0.531 |
| 2012 – 2018 | 12.356 | 8.634 | 1.431 | 0.479 |
| 2014 – 2016 | -0.173 | 8.027 | -0.022 | 1.000 |
| 2014 – 2018 | 0.529 | 8.027 | 0.066 | 1.000 |
| 2016 – 2018 | 0.701 | 8.037 | 0.087 | 1.000 |

*Porites rus*

| **Years** | **Estimate** | **St. Error** | **z-value** | **p-value** |
| --- | --- | --- | --- | --- |
| 2012 – 2014 | -22.332 | 6.299 | -3.545 | 0.002 |
| 2012 – 2016 | -27.203 | 5.539 | -4.911 | <0.001 |
| 2012 – 2018 | -21.945 | 6.299 | -3.484 | 0.819 |
| 2014 – 2016 | -4.872 | 5.595 | -.0871 | 0.819 |
| 2014 – 2018 | 0.387 | 6.293 | 0.061 | 1.000 |
| 2016 – 2018 | 5.259 | 5.595 | 0.940 | 0.782 |

**Macroalgae taxa**

All macroalgae

| **Years** | **Estimate** | **St. Error** | **z-value** | **p-value** |
| --- | --- | --- | --- | --- |
| **2012 – 2014** | **16.797** | **4.976** | **3.375** | **0.004** |
| **2012 – 2016** | **21.383** | **4.976** | **4.297** | **<0.001** |
| **2012 – 2018** | **14.629** | **4.976** | **2.940** | **0.018** |
| 2014 – 2016 | 4.586 | 4.976 | 0.922 | 0.793 |
| 2014 – 2018 | -2.168 | 4.976 | -0.436 | 0.972 |
| 2016 – 2018 | -6.753 | 4.976 | -1.357 | 0.526 |

*Halimeda*

| **Years** | **Estimate** | **St. Error** | **z-value** | **p-value** |
| --- | --- | --- | --- | --- |
| 2012 – 2014 | **14.788** | 5.82 | 2.541 | 0.053 |
| **2012 – 2016** | **22.536** | **5.82** | **3.872** | **<0.001** |
| **2012 – 2018** | **16.217** | **5.82** | **2.786** | **0.028** |
| 2014 – 2016 | 7.748 | 5.82 | 1.331 | 0.543 |
| 2014 – 2018 | 1.429 | 5.82 | 0.245 | 0.995 |
| 2016 – 2018 | -6.319 | 5.82 | 1.086 | 0.698 |

*Lobophora*

| **Years** | **Estimate** | **St. Error** | **z-value** | **p-value** |
| --- | --- | --- | --- | --- |
| 2012 – 2014 | 1.963 | 2.938 | 0.668 | 0.909 |
| 2012 – 2016 | -1.102 | 2.938 | -0.375 | 0.982 |
| 2012 – 2018 | -1.533 | 2.938 | -0.522 | 0.954 |
| 2014 – 2016 | -3.065 | 2.938 | -1.043 | 0.724 |
| 2014 – 2018 | -3.495 | 2.938 | -1.190 | 0.633 |
| 2016 – 2018 | -0.431 | 2.938 | -0.147 | 0.999 |

**Other benthic taxa**

CCA

| **Years** | **Estimate** | **St. Error** | **z-value** | **p-value** |
| --- | --- | --- | --- | --- |
| 2012 – 2014 | 2.220 | 2.336 | 0.951 | 0.777 |
| 2012 – 2016 | 4.272 | 2.336 | 1.829 | 0.260 |
| 2012 – 2018 | 3.755 | 2.336 | 1.608 | 0.374 |
| 2014 – 2016 | 2.051 | 2.336 | 0.878 | 0.816 |
| 2014 – 2018 | 1.534 | 2.336 | 0.657 | 0.913 |
| 2016 – 2018 | -0.517 | 2.336 | -0.221 | 0.996 |

Cyanobacteria

| **Years** | **Estimate** | **St. Error** | **z-value** | **p-value** |
| --- | --- | --- | --- | --- |
| 2012 – 2014 | 1.491 | 2.592 | 0.575 | 0.940 |
| 2012 – 2016 | 0.122 | 2.592 | 0.047 | 1.000 |
| 2012 – 2018 | 3.286 | 2.592 | 1.268 | 0.584 |
| 2014 – 2016 | -1.369 | 2.592 | -0.528 | 0.952 |
| 2014 – 2018 | 1.795 | 2.592 | 0.692 | 0.900 |
| 2016 – 2018 | 3.164 | 2.592 | 1.221 | 0.614 |

Rubble

| **Years** | **Estimate** | **St. Error** | **z-value** | **p-value** |
| --- | --- | --- | --- | --- |
| 2012 – 2014 | 3.139 | 1.807 | 1.737 | 0.304 |
| 2012 – 2016 | 3.945 | 1.807 | 2.183 | 0.128 |
| 2012 – 2018 | 1.009 | 1.807 | 0.558 | 0.944 |
| 2014 – 2016 | 0.805 | 1.807 | 0.446 | 0.971 |
| 2014 – 2018 | -2.130 | 1.807 | -1.179 | 0.640 |
| 2016 – 2018 | -2.954 | 1.807 | -1.624 | 0.365 |

Sand

| **Years** | **Estimate** | **St. Error** | **z-value** | **p-value** |
| --- | --- | --- | --- | --- |
| 2012 – 2014 | 3.659 | 3.552 | 1.030 | 0.732 |
| 2012 – 2016 | 0.233 | 3.552 | 0.065 | 1.000 |
| 2012 – 2018 | -0.881 | 3.552 | -0.248 | 0.995 |
| 2014 – 2016 | -3.436 | 3.552 | -0.965 | 0.770 |
| 2014 – 2018 | -4.540 | 3.552 | -1.278 | 0.577 |
| 2016 – 2018 | -1.114 | 3.552 | -0.314 | 0.989 |

Soft coral

| **Years** | **Estimate** | **St. Error** | **z-value** | **p-value** |
| --- | --- | --- | --- | --- |
| 2012 – 2014 | -0.097 | 0.096 | -1.005 | 0.746 |
| 2012 – 2016 | -0.101 | 0.096 | -1.049 | 0.720 |
| 2012 – 2018 | -0.046 | 0.096 | -0.480 | 0.964 |
| 2014 – 2016 | -0.004 | 0.096 | -0.044 | 1.000 |
| 2014 – 2018 | 0.050 | 0.096 | 0.525 | 0.953 |
| 2016 – 2018 | 0.055 | 0.096 | 0.569 | 0.941 |

Sponges

| **Years** | **Estimate** | **St. Error** | **z-value** | **p-value** |
| --- | --- | --- | --- | --- |
| 2012 – 2014 | 0.097 | 0.107 | 0.899 | 0.805 |
| 2012 – 2016 | 0.101 | 0.107 | 0.939 | 0.784 |
| 2012 – 2018 | 0.046 | 0.107 | 0.429 | 0.973 |
| 2014 – 2016 | 0.004 | 0.107 | 0.040 | 1.000 |
| 2014 – 2018 | -0.050 | 0.107 | -0.470 | 0.966 |
| 2016 – 2018 | -0.054 | 0.107 | -0.509 | 0.957 |

Turf algae

| **Years** | **Estimate** | **St. Error** | **z-value** | **p-value** |
| --- | --- | --- | --- | --- |
| 2012 – 2014 | 15.844 | 6.298 | 2.516 | 0.057 |
| **2012 – 2016** | **23.062** | **6.298** | **3.662** | **0.001** |
| 2012 – 2018 | 14.061 | 6.298 | 2.232 | 0.114 |
| 2014 – 2016 | 7.218 | 6.298 | 1.146 | 0.661 |
| 2014 – 2018 | -1.784 | 6.298 | -0.283 | 0.992 |
| 2016 – 2018 | -9.002 | 6.298 | -1.429 | 0.481 |
