# Supplementary material for: Coral reefs in the Gilbert Islands of Kiribati: Resistance, resilience, and recovery after more than a decade of multiple stressors
Source: PLoS One. 2021 Aug 11;16(8):e0255304. doi: 10.1371/journal.pone.0255304 (PMC8357116; doi:10.1371/journal.pone.0255304)
Supplement: S5 Table — Includes Welch’s ANOVA of size-frequency statistics between years, and KS test results comparing size frequency distributions across years within each atoll. (DOCX) [file pone.0255304.s005.docx]

**S5 Table. Results of Size Frequency statistical analyses.**

**Results including all years**
 **Welch's ANOVA of size-frequency statistics between years, with significant results bolded.**

| **Taxa** | **Mean^1^** | | | **Coefficient of Variation** | | | **Kurtosis** | | | **Skewness** | | |
| --- | --- | --- | --- | --- | --- | --- | --- | --- | --- | --- | --- | --- |
|  | **F** | **p** | **df^2^** | **F** | **p** | **df^2^** | **F** | **p** | **df^2^** | **F** | **p** | **df^2^** |
| *Acropora* | 1.35 | 0.47 | 1.69 | 0.58 | 0.68 | 1.99 | -- | -- | -- | -- | -- | -- |
| Favids | 2.06 | 0.36 | 1.81 | 1.57 | 0.41 | 2.05 | 0.24 | 0.86 | 1.85 | 0.17 | 0.91 | 1.71 |
| *Heliopora* | 1.89 | 0.39 | 1.71 | 1.07 | 0.51 | 2.09 | 7.90 | 0.13 | 1.83 | 9.36 | 0.10 | 2.03 |
| *Montipora* | 4.37 | 0.21 | 1.86 | **37.23** | **0.04** | **1.67** | -- | -- | -- | 0.74 | 0.63 | 1.77 |
| *Pocillopora* | 1.77 | 0.41 | 1.70 | 6.97 | 0.12 | 2.12 | **40.20** | **0.04** | **1.68** | 5.36 | 0.15 | 2.08 |
| Massive *Porites* | 7.63 | 0.13 | 1.82 | **139.35** | **0.01** | **2.02** | 0.55 | 0.70 | 1.77 | 0.95 | 0.57 | 1.70 |

^1^ Mean size in cm

^2^ df is the denominator degrees of freedom. Numerator degrees of freedom equals 1 for all tests.

**Kolmogorov-Smirnov (KS) test results comparing size distributions across years within each atoll, with Bonferroni correction. Results that are significant are in bold.**

|  | **All years** | | | | | | **2012 & 2018** | | | | | |
| --- | --- | --- | --- | --- | --- | --- | --- | --- | --- | --- | --- | --- |
|  | **Abaiang** | | | **Tarawa** | | | **Abaiang** | | | **Tarawa** | | |
| **Taxa** | **D** | **p** | **p-adj** | **D** | **p** | **p-adj** | **D** | **p** | **p-adj** | **D** | **p** | **p-adj** |
| *Acropora* | 0.45 | 0.61 | 1.00 | 0.22 | 0.66 | 1.00 | 0.45 | 0.07 | 0.44 | 0.32 | 0.43 | 1.00 |
| Favids | **0.82** | **<0.01** | **<0.01** | 0.14 | 0.82 | 1.00 | **0.69** | **<0.01** | **<0.01** | 0.15 | 0.70 | 1.00 |
| *Heliopora* | **0.51** | **<0.01** | **<0.01** | 0.25 | 0.03 | 0.25 | **0.74** | **<0.01** | **<0.01** | **0.51** | **<0.01** | **<0.01** |
| *Montipora* | 0.58 | 0.39 | 1.00 | 0.33 | 0.89 | 1.00 | 0.36 | 0.83 | 1.00 | 0.53 | 0.17 | 0.87 |
| *Pocillopora* | 0.23 | 0.43 | 1.00 | 0.19 | 0.40 | 1.00 | **0.70** | **<0.01** | **<0.01** | **0.39** | **<0.01** | **<0.01** |
| Massive *Porites* | **0.53** | **<0.01** | **<0.01** | 0.61 | 0.14 | 1.00 | **0.76** | **<0.01** | **<0.01** | 0.49 | 0.18 | 0.87 |
