# Supplementary material for: Coral reefs in the Gilbert Islands of Kiribati: Resistance, resilience, and recovery after more than a decade of multiple stressors
Source: PLoS One. 2021 Aug 11;16(8):e0255304. doi: 10.1371/journal.pone.0255304 (PMC8357116; doi:10.1371/journal.pone.0255304)
Supplement: Attachment — Submitted filename: ResponsetoReviewers.docx [file pone.0255304.s007.docx]

15 June, 2021

Re: PONE-D-21-04308, “Coral reefs in the Gilbert Islands of Kiribati: Resistance, resilience, and recovery after more than a decade of multiple stressors”

Dear Dr. Guest,

Thank you for the detailed and helpful feedback and the two anonymous reviews you have shared with us for our recent manuscript submission to PLOS ONE. On behalf of my co-authors, I am submitting a revised manuscript that responds to the very helpful suggestions and concerns raised by the reviewers.

In response to the reviewer’s comments, we have removed the acronym ‘ASS’ (although please note that we did not invent this acronym; it was used in the literature we cited) and removed the first full table to the supplementary materials, have integrated the future directions section into the discussion (with less detail), and have added a paragraph to explain the importance of these results to coral reefs outside of the Gilbert Islands.

I am including my responses to each of the comments below (the original comment is in regular text and my response is in italics). I have highlighted where we made changes in the manuscript to address the comments and have also uploaded two versions of the revised manuscript (one that tracks the changes, and one that does not), per your instruction.

Thank you again for your response and suggestions, and for the opportunity to respond. We look forward to hearing back from you.

Sincerely,

Sara E. Cannon, M.Sc., Ph.D. Candidate

University of British Columbia

Reviewer #1: Cannon et al. have made an adequate attempt to revise their manuscript, and this version is better than the first. Overall, the science message is fine and the results support the conclusion. The text remains parochial with limited appeal to readers who don’t work on coral reefs or even those who don’t have interest in the Gilbert Islands, and the manuscript remains far longer than it needs to be. This sort of material does not need 7 tables (including 2 full page versions) to summarize and present the pertinent data, and further consideration should be given to moving these to supplemental material, or replacing them with a more synthetic data summary. The “Future Directions” and “Conclusions” statements should be dropped from the manuscript since they either contain material that is not relevant to the questions/hypothesis guiding this work or are duplicative. Let’s not propagate the use of “ASS” as an acceptable acronym in coral reef literature.

*Thank you for your helpful feedback. We have moved the first table with all of our site information to the supplementary materials to further reduce the number of tables in the manuscript and have added more information in the introduction and the discussion sections about why this work is important for coral reefs in other parts of the world (although we understand that it may not be relevant to people working outside of coral reef ecology).*

*Specifically, we edited the second paragraph of the abstract to read:*

*“The coral reefs of the Republic of Kiribati’s Gilbert Islands are exposed to frequent heat stress caused by central-Pacific type El Niño events, and may provide a glimpse into the future of coral reefs in other parts of the world, where the frequency of heat stress events will likely increase due to climate change.”*

*We also added a similar message to lines 45-50 of the abstract, which now read:*

*“These findings provide a rare glimpse at the future of coral reefs around the world and the ways they may be affected by climate change, which may allow scientists to better predict how other reefs will respond to increasing heat stress events across gradients of local human disturbance. We end with suggestions for future research that would address gaps in our analyses, with implications for coral reefs and the people depending on them, both locally and globally.”*

*In the introduction, we added at line 81:*

*“The coral reefs of Tarawa Atoll and its less populated neighbor Abaiang Atoll in the Republic of Kiribati provide a unique opportunity to investigate the role of chronic human disturbances on coral reef recovery from acute disturbances, and could also serve as examples of the ways that reefs in other parts of the world may respond to increasing frequencies of climate-driven heat stress events in the future.”*

*We also added, at line 184:

“Our findings provide a rare glimpse at how coral reefs around the world may respond to the increasing frequencies of heat stress events across gradients of local human disturbance and could provide important lessons to guide the future management of coral reef resources in the face of climate change.”*

*Finally, in the conclusion, we removed the sections “Future Directions” and “Conclusions” per your suggestion, and added a paragraph to summarize the importance of this work for reefs in other places in the future, beginning at line 956:

“Coral reefs in the Gilbert Islands have experienced years with prolonged heat stress more frequently than 99% of the world’s coral reefs [19], but this may change in the future; other reefs will likely experience more heat stress going forward, given that climate change-driven global coral bleaching events have increased in frequency and are expected to continue increasing in the due to climate change [93]. Reefs in the Gilbert Islands could therefore provide a rare glimpse into what reefs may look like in the future while also accounting for a gradient of local human impacts. We hope this work, coupled with further investigations into the potential trade-offs associated with locally degraded but climate-resistant reefs such as found in Tarawa versus reefs with more long-term ability to recover but with less resistance such as those in Abaiang, will provide novel information that is important for the future management of coral reef resources. Specifically, these findings may help to predict the ways that climate change will affect the millions of people around the world who depend on coral reefs, so that they may prepare for the future.”*

*We have also removed the acronym ASS per your suggestion and agree that it is less than ideal, although please note that it was not our invention (the acronym is already in use in several of the publications that we cited).*

Reviewer #2: Overall, I think that the authors did a good job of reorganizing the manuscript and clarifying their questions and hypotheses. I have just a few small comments on the revised version, but after they are addressed, I think this manuscript is ready for publication.

Edits
Line 23 - 'year' should be 'years'
Line 28 - repeated phrase
Line 113 - 'sewerage' should be 'sewage'?
Reference entry errors - lines 474, 483,
Line 688 (and following) - I think you should just use the whole phrase 'alternative stable state', as I just couldn't get past repeatedly reading 'ASS'.
Lines 762-766 - It feels a little weird to include these specific numbers without the associated error, especially since the errors overlap in at least some cases. I think you could leave it as is, but add in the errors to the numbers in parentheses; e.g., "(from 1.09% in 2014 to 2.90% in 2018)"

*Thank you for these suggested edits. We have made the changes in the manuscript.*

Lines 791-792 - I am a little hesitant about this, as it seems like you're using a lack of evidence to suggest that there aren't chronic disturbances in Abaiang. Although I know that Abaiang is much less populated than Tarawa, it still does have a human population, and so there could be disturbances that you aren't measuring. Please soften this statement to acknowledge that possibility.

*We appreciate you pointing this out and we agree that chronic disturbances likely do exist in Abaiang, although at much less intensity/severity than in Tarawa. We have updated the text to now read the following, on line 857-858: “We have no evidence that either of these two conditions currently exist in Abaiang.”*


Line 930 - repeated word
Line 935 - change 'what' to 'which'

*Thank you for your comments and suggested edits. We have removed this section from the manuscript per the suggestion of the other reviewer.*
